# SOX2 Inhibits Cuproptosis to Affect Cisplatin Resistance in Non‐Small Cell Lung Cancer by Activating the Wnt/ATP7B Signaling Pathway

**DOI:** 10.1002/kjm2.70286

**Published:** 2026-09-18

**Authors:** Kun Chen, Fu‐Mei He, Ying‐Hui Huang, Ting Fang, Yi‐Ping Zheng, Jian‐Feng Cai

**Affiliations:** ^1^ Pulmonary and Critical Medicine Nanping First Hospital Affiliated to Fujian Medical University Nanping Fujian China

**Keywords:** ATP7B, cisplatin resistance, cuproptosis, SOX2, Wnt pathway

## Abstract

Cisplatin (CDDP) resistance constitutes the principal clinical challenge in the treatment of advanced non‐small cell lung cancers (NSCLC). Disturbances in copper metabolism are closely linked with tumor drug resistance. SOX2, a pivotal stemness transcription factor (TF), holds an important role in chemotherapy resistance. However, whether it influences CDDP resistance by modulating cuproptosis remains unknown. SOX2 level was tested by qPCR and WB, and ATP7B level was evaluated by WB and flow cytometry. The oligomerization levels of FDX1, LIAS, and DLAT, together with Cu^2+^ levels, were determined by WB, confocal microscopy, and a Cu^2+^ colorimetric assay kit. The proliferation and apoptosis of NSCLC drug‐resistant cells were assessed with CCK‐8 and flow cytometry. SOX2 was significantly up‐regulated in CDDP‐resistant NSCLC cells, accompanied by decreased cuproptosis sensitivity. Silencing SOX2 in CDDP‐resistant cells increased cuproptosis sensitivity and apoptosis rate and reduced cell viability. These effects of SOX2 silencing on CDDP resistance in NSCLC cells were reversed by the cuproptosis inhibitor TTM. SOX2 up‐regulated ATP7B by activating the Wnt/β‐catenin signaling pathway. Silencing ATP7B or treatment with the Wnt pathway inhibitor LF3 attenuated SOX2 overexpression‐induced promoting effect on CDDP resistance and suppressive effect on cuproptosis in NSCLC. In summary, this study reveals a novel mechanism wherein SOX2 activates the Wnt/β‐catenin signaling pathway to upregulate ATP7B expression, thereby suppressing cuproptosis and ultimately driving cisplatin resistance in NSCLC. These findings provide potential therapeutic targets for reversing chemoresistance in NSCLC.

## 1 Introduction

Chemotherapy is the standard adjuvant treatment for early‐stage non‐small cell lung cancer (NSCLC); however, postoperative cisplatin (CDDP)‐based chemotherapy improves the 5‐year survival rate by only 4%–5% [1], and drug‐resistant relapse remains the major clinical challenge [2]. A study has revealed that intracellular copper ion (Cu^2+^) homeostasis imbalance is closely linked to chemosensitivity [3]. CTR1 assumes an important role in increasing cellular CDDP uptake and sensitivity [4]. Moreover, excessive Cu^2+^ accumulation can trigger a novel cell death modality—cuproptosis—thereby suppressing chemoresistance in malignant tumors such as NSCLC [5] and ovarian cancer [6]. These findings suggest that an in‐depth dissection of the mechanisms underlying cuproptosis will provide new targeting strategies for NSCLC therapy.

As a key copper‐transporting protein, ATP7B maintains intracellular copper homeostasis through active copper efflux; when ATP7B is overexpressed, the intracellular free copper concentration is reduced, directly repressing the core trigger of cuproptosis [7]. In NSCLC, ATP7B inhibition can induce cuproptosis and enhance tumor cell apoptosis [5]. Additionally, miR‐133a‐5p can promote cuproptosis by targeting ATP7B, a mechanism that has been verified in hepatocellular carcinoma [8]. In view of ATP7B's function in regulating cuproptosis, it has become a novel target for potentiating chemotherapy efficacy in prostate cancer [9]. Notably, the β‐catenin/TCF4 transcription complex can directly bind to the ATP7B promoter and up‐regulate its expression, revealing the pivotal role of the Wnt/β‐catenin in modulating cuproptosis sensitivity via ATP7B [10]. Nevertheless, whether the Wnt/β‐catenin–ATP7B axis participates in the molecular mechanisms of CDDP resistance in NSCLC remains to be elucidated.

SOX2, a core TF for embryonic development and stem‐cell pluripotency, is aberrantly highly expressed in NSCLC cancer stem cells (CSCs) and endows cells with self‐renewal, metastatic, and drug‐resistant capacities [11, 12, 13]. Moreover, numerous studies have indicated a complex bidirectional regulatory relationship between SOX2 and the Wnt/β‐catenin signaling. SOX2 can up‐regulate β‐catenin and induce its nuclear translocation, thereby triggering the Wnt/β‐catenin signaling [14, 15], whereas Wnt activation can in turn up‐regulate SOX2 expression [16], forming a positive‐feedback loop that maintains stemness. This phenomenon suggests that the SOX2–Wnt axis may participate in the molecular regulation of Cu^2+^ homeostasis and death resistance by controlling downstream target genes.

The present work aimed to clarify the regulatory role of SOX2 in CDDP resistance of NSCLC and its underlying molecular mechanisms. SOX2 up‐regulated ATP7B by triggering the Wnt/β‐catenin signaling, thereby suppressing cuproptosis and ultimately reinforcing CDDP resistance in NSCLC cells. As the first to reveal the pivotal role of the SOX2/Wnt/β‐catenin/ATP7B signaling axis in modulating CDDP sensitivity in NSCLC cells, this work offers new theoretical evidence for understanding the mechanisms underlying chemoresistance in lung cancer (LC).

## 2 Methods

### 2.1 Reagents and Antibodies

Tetrathiomolybdate (TTM) and the Wnt signaling pathway inhibitor LF3 were bought from MedChemExpress (MCE, USA); the concentrations for cell treatment were 20 μM and 10 μM, respectively. Elesclomol‐Cu (ES‐Cu) was bought from MedChemExpress (MCE, USA). The concentration for cell treatment was 20 nM.

Antibodies included SOX2 (ab137385, Abcam, UK), FDX1 (ab108257, Abcam, UK), LIAS (ab96302, Abcam, UK), ATP7B (ab124973, Abcam, UK), β‐catenin (ab68183, Abcam, UK), GSK‐3β (51065‐1‐AP, Proteintech, USA), p‐GSK‐3β (#9336, Cell Signaling, USA), Caspase 3 (ab32351, Abcam, UK), Cleaved Caspase 3 (ab32042, Abcam, UK), DLAT (13426‐1‐AP, Proteintech, USA), GAPDH (10494‐1‐AP, Proteintech, USA), and secondary IgG (ab6759 or ab150077, Abcam, UK).

### 2.2 Cell Cultivation

The LC cell line A549 and the CDDP‐resistant cell line A549‐DDP were bought from Procell (China) and were authenticated by STR profiling. A549 cells were cultivated in Ham's F‐12 K medium (Thermo Fisher Scientific, USA) supplemented with 10% FBS and 1% P/S. A549‐DDP cells were kept in Ham's F‐12 K medium (Thermo Fisher Scientific, USA) with 10% FBS, 1 μg/mL DDP, and 1% P/S. The incubator parameters were set for cell incubation (37°C, 5% CO_2_, saturated humidity). Subsequent experiments were continued when the cells were in good condition.

### 2.3 Cell Transfection

Small interfering (si) RNAs targeting SOX2 (si‐SOX2) and ATP7B (si‐ATP7B), as well as the corresponding negative control (si‐NC), pcDNA3.1‐SOX2, and empty vector (pcDNA3.1‐NC), were constructed by RiboBio (China). The indicated siRNAs and plasmids were transfected into A549‐DDP cells for 48 h with Lipofectamine 3000 (Invitrogen).

### 2.4 Cell Viability Assay

After cell cultivation from different treatment groups, 10 μL CCK‐8 reagent was introduced into per 100 μL medium, incubated in a 37°C incubator for 2 h. Absorbance of each well (450 nm) was examined with a microplate reader. The experiment was repeated three times. For IC₅₀ determination, cells were exposed to CDDP for 2 days (2.5, 5, 15, 30, and 50 μg/mL). IC_50_ values were examined based on cell viability results.

### 2.5 qPCR Assay

RNA was isolated from cells with Trizol (Invitrogen, USA). cDNA was synthesized with the HiScript III 1st Strand cDNA Synthesis Kit (Vazyme, China) with a total of 500 ng RNA. qPCR was undertaken on the 7500 Real‐Time PCR System (Applied Biosystems, USA) with the Taq Pro Universal SYBR qPCR Master Mix (Vazyme, China) to determine the indicated RNA levels. Primer sequences are displayed in Table 1. GAPDH was the normalization reference.

**TABLE 1:** Primer set for qRT‐PCR.

Gene	Primer sequence (5′ → 3′)
SOX2 (NCBI accession number: NM_003106.4)	F: TACAGCATGTCCTACTCGCAG
	R: GAGGAAGAGGTAACCACAGGG
GAPDH	F: GGAGCGAGATCCCTCCAAAAT R: GGCTGTTGTCATACTTCTCATGG

### 2.6 Western Blot (WB) Assay

A549 or A549‐DDP cells from each group were homogenized and lysed in RIPA lysis buffer for 30 min at low temperature; proteins were extracted. Protein concentration was determined with a protein quantification kit. Protein samples were separated by SDS‐PAGE, transferred to PVDF membranes, blocked with 5% skim milk for 1 h, and incubated with primary antibodies against SOX2, FDX1, LIAS, ATP7B, β‐catenin, GSK‐3β, p‐GSK‐3β, and GAPDH overnight at 4°C. Membranes were incubated with secondary antibodies for 4 h at room temperature (RT). Bands were visualized with ECL chemiluminescence detection reagents on the ChemiScope 6000 chemiluminescence and imaging system (Clinx, China).

### 2.7 IF Assay

A549‐DDP cells were immobilized in 4% paraformaldehyde (PFA) for 10 min, permeabilized with 0.1% Triton X‐100 for 10 min, and blocked with 3% BSA for 30 min. Cells were incubated with DLAT antibody (1:500) at 4°C overnight, rinsed three times with 0.1% PBS, and then incubated with fluorescence‐labeled secondary IgG (37°C, 1 h). Finally, slides were mounted with mounting medium containing DAPI and imaged with a fluorescence microscope. For mitochondrial staining, cells were incubated with 200 nM MitoTracker Red CMXRos (Cell Signaling, USA) for 30 min before fixation.

### 2.8 Flow Cytometry (FCM) Analysis

A549‐DDP cells from each group were rinsed three times with pre‐cooled PBS, resuspended in 195 μL binding buffer, and incubated with 5 μL Annexin V/FITC solution (Beyotime, China) for 10 min without lights, followed by 10 μL PI for an additional 25 min of dark incubation. Cells were kept on ice and protected from light prior to FCM analysis. Total apoptosis rate (%) was calculated as the sum of early and late apoptosis rates.

For intracellular ATP7B detection, A549‐DDP cells (1 × 10^6^) were digested, rinsed, immobilized with 4% PFA at RT for 15 min, centrifuged to remove fixative, permeabilized with 0.1% Triton X‐100 for 15 min at RT, and incubated with ATP7B primary antibody at 4°C overnight. After centrifugation and washing, fluorescence‐labeled secondary IgG was introduced for 30 min of dark incubation at RT. Cells were resuspended in FCM staining buffer and analyzed by FCM.

### 2.9 Cu^2+^ Colorimetric Assay

Following the instructions of the Cu^2+^ Colorimetric Assay Kit (Elabscience, China), standards and samples were put into a 96‐well plate. Then, 50 μL chromogenic working solution was put into each well. After incubation at 37°C for 5 min, the OD value (580 nm) was tested with a microplate reader to determine Cu^2+^ concentration.

### 2.10 Data Analysis

Statistical analysis was processed with SPSS 23.0 and GraphPad Prism version 10.1.2. Comparisons between two groups were completed with *t*‐tests, and multiple groups were compared via one‐way ANOVA. Data are presented as mean ± SD (*p* < 0.05: statistically significant).

## 3 Results

### 3.1 SOX2 Is Highly Expressed and Cuproptosis Is Suppressed in CDDP‐Resistant NSCLC Cells

**FIGURE 1:**
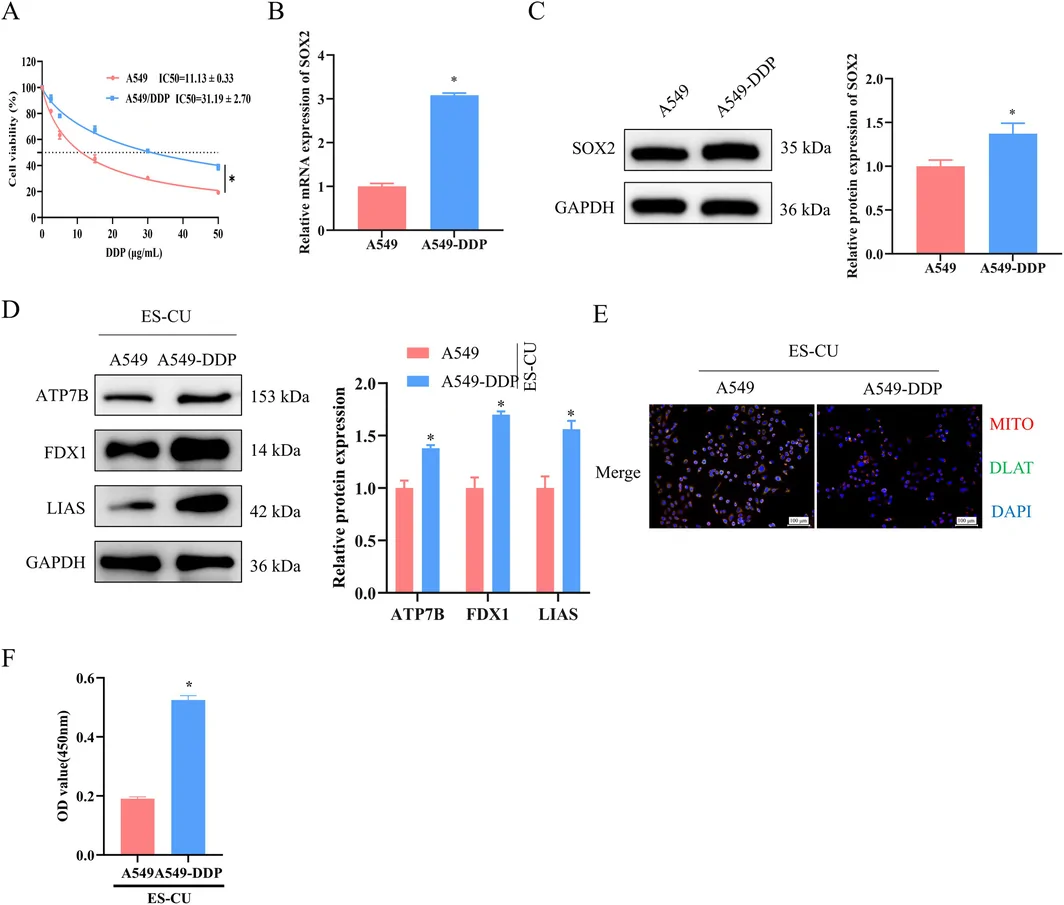
SOX2 is highly expressed and cuproptosis is suppressed in CDDP‐resistant NSCLC cells (A) CCK‐8 assay for DDP IC_50_ detection in A549 and A549/DDP cells. (B, C) SOX2 expression determined by qPCR and WB. (D–F) After co‐culturing A549 and A549/DDP cells with 20 nM ES‐CU, the expression of ATP7B, FDX1, LIAS (D), DLAT aggregation degree (E), and cell viability (F) were analyzed. *p < 0.05.

The IC_50_ value of A549‐DDP (IC_50_ = 11.13 ± 0.33) cells was higher than that of A549 (IC_50_ = 31.19 ± 2.70) cells, indicating CDDP resistance (Figure 1A). SOX2 expression was higher in CDDP‐resistant A549 cells (Figure 1B,C). These data indicate upregulated SOX2 expression in CDDP‐resistant NSCLC cells. Elesclomol‐Cu is a complex formed by Elesclomol and Cu^2+^, which is capable of inducing cuproptosis. A549 and A549‐DDP cells were treated with 20 nM ES‐CU. Compared with A549 cells, A549‐DDP cells exhibited greatly increased ATP7B, FDX1, and LIAS levels (Figure 1D). DLAT is a key target protein of cuproptosis. Copper ions directly bind to DLAT and induce its oligomerization, which subsequently triggers cell death [17]. Accordingly, we detected the aggregation level of DLAT via immunofluorescence. The results revealed markedly reduced DLAT aggregation in A549‐DDP cells (Figure 1E). Meanwhile, the viability of CDDP‐resistant A549 cells was higher than that of A549 cells (Figure 1F). Cuproptosis sensitivity is greatly reduced in CDDP‐resistant NSCLC cells.

### 3.2 SOX2 Silencing Enhances Cuproptosis Sensitivity and Reduces CDDP Resistance in NSCLC

**FIGURE 2:**
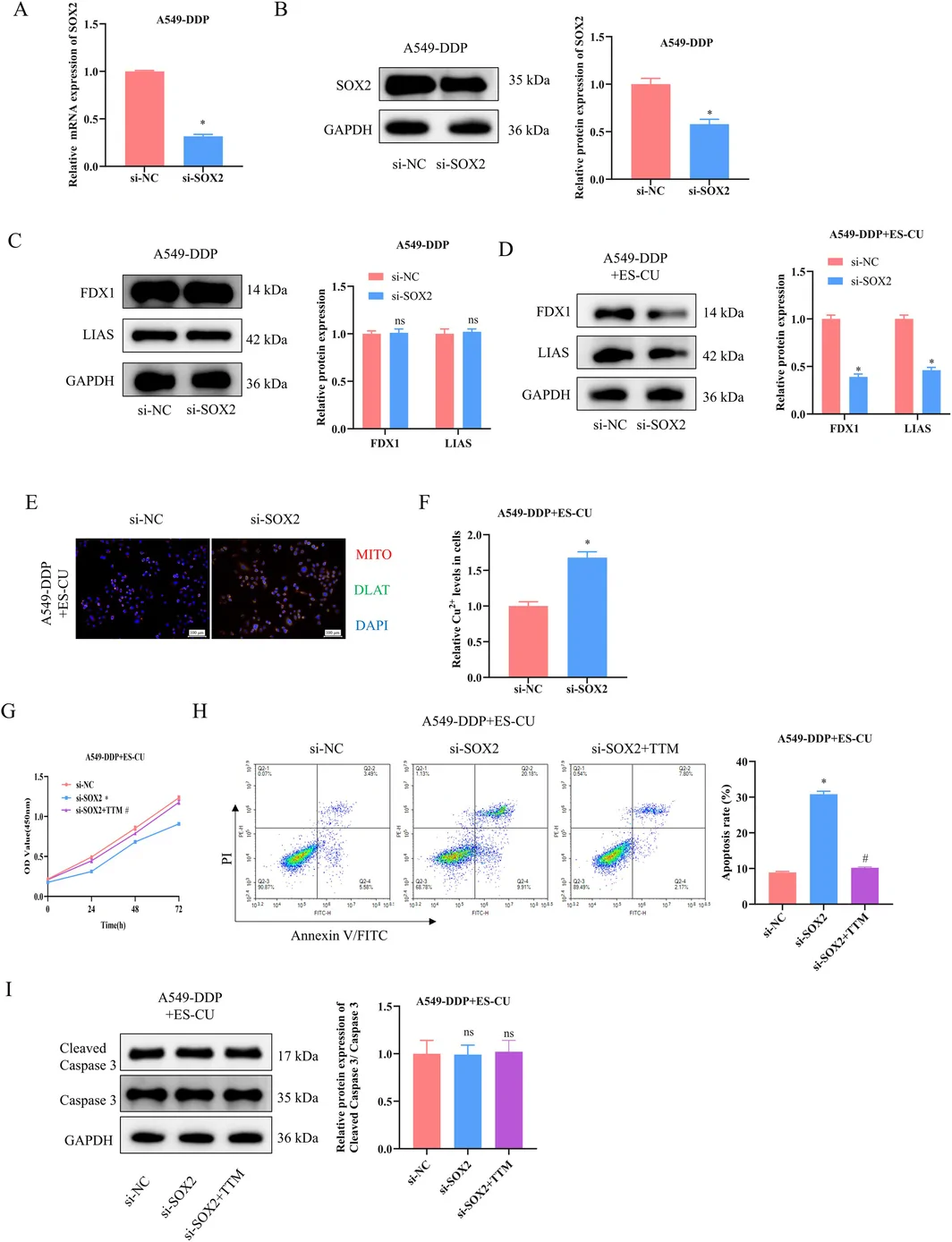
SOX2 modulates cuproptosis to influence CDDP resistance in NSCLC cells. (A–C) A549‐DDP cells were transfected with si‐NC or si‐SOX2; SOX2 mRNA (A), protein (B), and FDX1/LIAS expression (C) were detected. (D–F) After co‐culture of A549‐DDP and ES‐CU, FDX1/LIAS protein expression (D), DLAT oligomerization (E), and intracellular Cu^2+^ level (F) were assessed. (G‐H) A549‐DDP cells were divided into si‐NC, si‐SOX2, and si‐SOX2 + TTM groups; cell viability (G) and apoptosis rate (H) were measured after ES‐CU treatment. (H) WB was used to detect the expression levels of apoptosis‐related proteins, cleaved‐caspase 3 and caspase 3. *p < 0.05 versus si‐NC; #*p* < 0.05 versus si‐SOX2.

We further probed into the effect of SOX2 silencing on cuproptosis in NSCLC cells. Transfection of si‐SOX2 into A549‐DDP cells significantly downregulated SOX2 at both mRNA and protein levels (Figure 2A,B). Notably, SOX2 silencing alone did not alter cuproptosis‐related protein expression (Figure 2C). However, under cuproptosis‐inducing conditions (co‐cultivation of A‐549‐DDP and ES‐CU), SOX2 knockdown led to downregulated FDX1 and LIAS expression, along with enhanced DLAT aggregation and intracellular Cu^2+^ levels (Figure 2D–F). To verify the role of SOX2‐regulated cuproptosis in CDDP resistance, we treated SOX2‐silenced A549‐DDP cells with the cuproptosis inhibitor TTM. In CCK‐8 and FCM assays, SOX2 silencing inhibited cell proliferation and induced apoptosis, but these effects were reversed by TTM treatment (Figure 2G,H). WB results showed no significant changes in the expression of caspase‐3 and cleaved caspase‐3 among all groups (Figure 2I). Thus, SOX2 silencing suppresses CDDP resistance in NSCLC cells by inducing cuproptosis.

### 3.3 SOX2 Upregulates ATP7B via Triggering the Wnt Signaling Pathway

**FIGURE 3:**
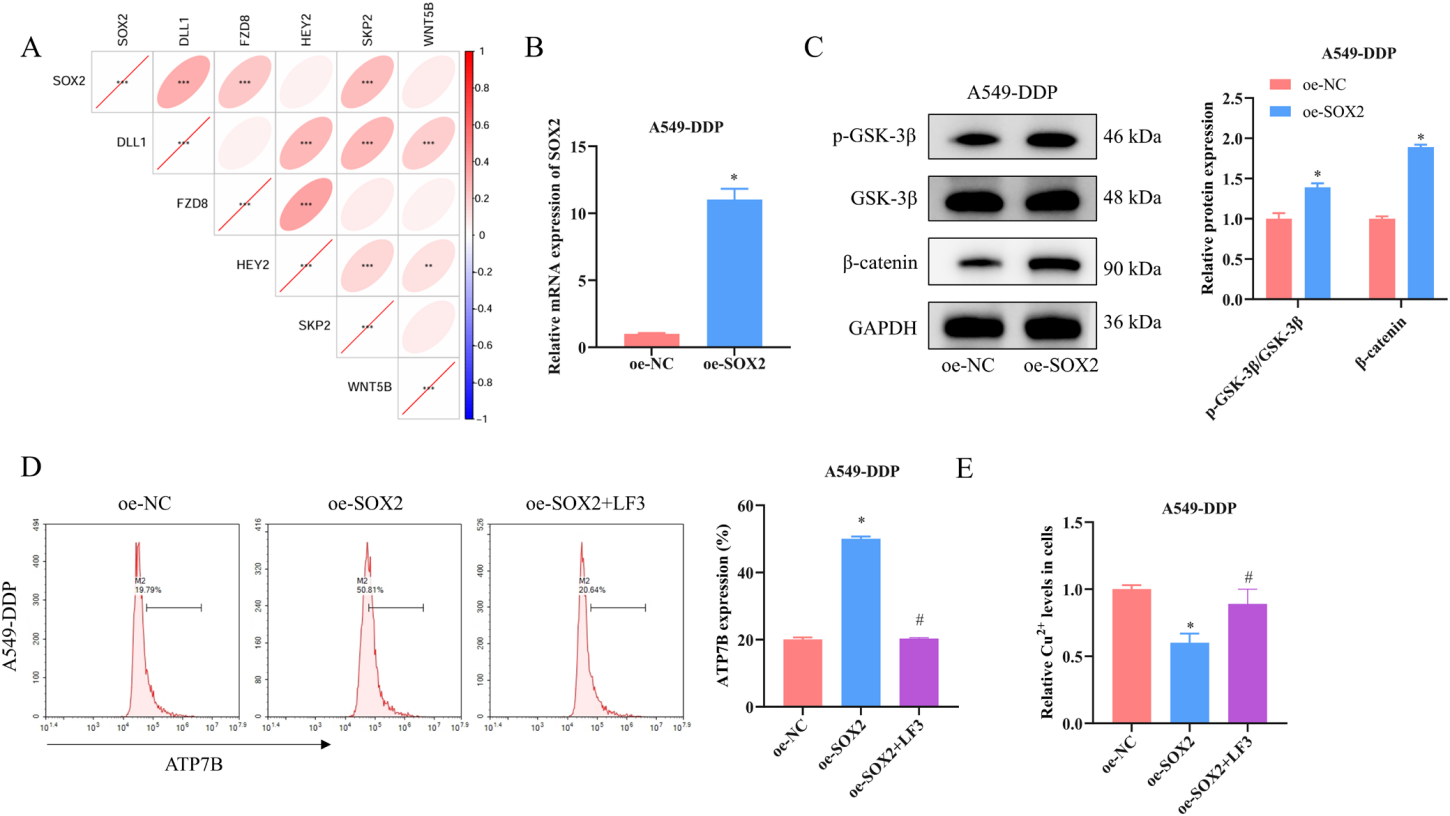
SOX2 up‐regulates ATP7B transcription via Wnt/β‐catenin signaling. (A) Correlation between SOX2 and Wnt downstream targets in the LUAD‐TCGA database. (B) SOX2 mRNA expression in oe‐NC and oe‐SOX2 A549‐DDP cells. (C) WB for GSK‐3β, p‐GSK‐3β, and β‐catenin. (D) A549‐DDP cells were divided into oe‐NC, oe‐SOX2, and oe‐SOX2 + LF3 groups, and FCM assessed ATP7B expression. (E) Intracellular Cu^2+^ levels in oe‐NC, oe‐SOX2, and oe‐SOX2 + LF3 groups. ***p < 0.001; *p < 0.05 versus oe‐NC; #*p* < 0.05 versus oe‐SOX2.

To elucidate the molecular mechanism by which SOX2 regulates cuproptosis, we first performed correlation analysis based on the LUAD‐TCGA database and discovered that SOX2 expression was significantly positively linked with key Wnt/β‐catenin signaling effectors DLL1 [18], FZD8 [19], and SKP2 [20] (Figure 3A). Subsequently, SOX2 overexpression in A549‐DDP cells (Figure 3B) significantly activated the Wnt/β‐catenin pathway, as evidenced by elevated phosphorylated GSK‐3β levels and β‐catenin accumulation (Figure 3C). Former studies report that the β‐catenin/TCF4 transcription complex directly binds the ATP7B promoter [10] and that ATP7B overexpression boosts copper efflux to suppress cuproptosis [7]. We therefore further did intervention experiments with the β‐catenin/TCF4 interaction antagonist LF3. In FCM analysis, SOX2 overexpression greatly elevated ATP7B expression, whereas LF3 treatment partially reversed this effect (Figure 3D). Next, we detected intracellular Cu^2+^ levels in oe‐NC, oe‐SOX2 and oe‐SOX2 + LF3 groups using a Cu^2+^ detection kit. The results demonstrated that intracellular Cu^2+^ content was significantly decreased in the oe‐SOX2 group compared with the oe‐NC group, whereas treatment with the Wnt pathway inhibitor LF3 restored Cu^2+^ levels (Figure 3E). Collectively, the SOX2–Wnt/β‐catenin–ATP7B axis holds a central role in suppressing cuproptosis.

### 3.4 SOX2‐Mediated Wnt/ATP7B Signaling Suppresses Cuproptosis to Promote CDDP Resistance

**FIGURE 4:**
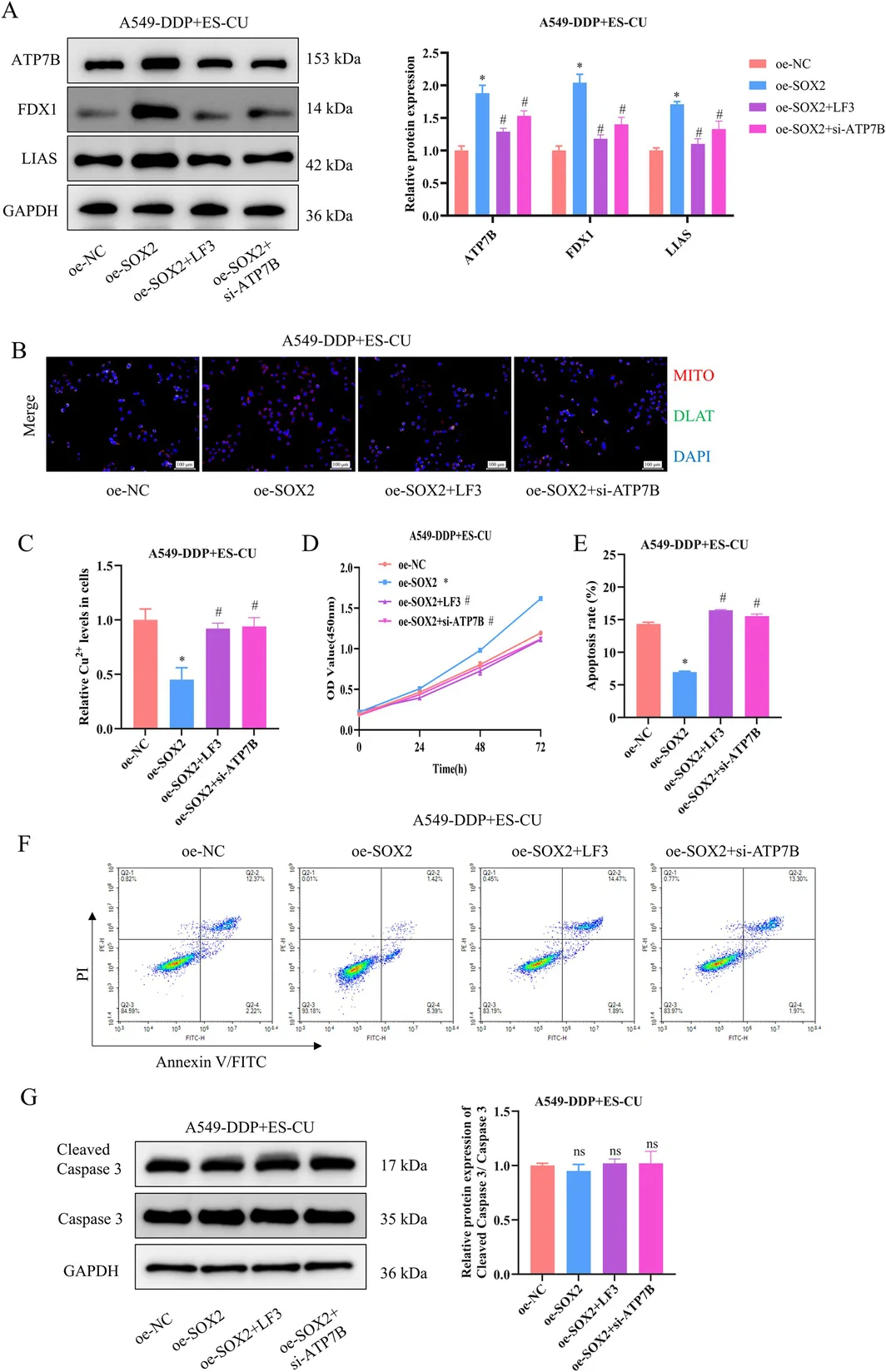
Blockade of Wnt/ATP7B signaling restores cuproptosis and reverses SOX2‐induced CDDP resistance. (A–C) A549‐DDP cells (oe‐NC, oe‐SOX2, oe‐SOX2 + LF3, oe‐SOX2 + si‐ATP7B) were co‐cultured with ES‐CU. ATP7B, FDX1, LIAS protein (A), DLAT oligomerization (B), and Cu^2+^ level (C) were analyzed; (D‐F) Cell viability (D) and apoptosis (E–F) were evaluated. (G) WB analysis was performed to examine the protein expression of apoptosis‐related proteins, caspase‐3 and cleaved caspase‐3. *p < 0.05 versus oe‐NC; #*p* < 0.05 versus oe‐SOX2.

To further figure out the linkage between SOX2 and ATP7B in regulating CDDP resistance, A549‐DDP cells were transfected with oe‐NC, oe‐SOX2, oe‐SOX2 + LF3, or oe‐SOX2 + si‐ATP7B. WB, IF staining, and Cu^2+^ colorimetric assays demonstrated that SOX2 overexpression up‐regulated ATP7B and attenuated cuproptosis, whereas either LF3 treatment or ATP7B silencing markedly weakened these effects (Figure 4A–C). Moreover, compared with the negative‐control group, SOX2 overexpression significantly enhanced cell viability and reduced apoptosis; these outcomes were substantially reversed when LF3 was added or ATP7B was silenced (Figure 4D–F). WB results indicated no significant differences in caspase‐3 and cleaved caspase‐3 levels across all groups (Figure 4G). Collectively, SOX2 activates the Wnt/ATP7B axis, suppresses cuproptosis, and thereby promotes CDDP resistance.

## 4 Discussion

Although the molecular basis of NSCLC chemoresistance has been investigated, identification of key regulatory targets remains a major challenge [21]. Cuproptosis, a newly recognized cell‐death modality, offers a novel therapeutic strategy for cancer [22]. Herein, we demonstrate that SOX2 drives CDDP resistance in NSCLC by triggering Wnt/β‐catenin signaling, upregulating ATP7B, and consequently repressing cuproptosis. This finding provides a theoretical framework for cuproptosis‐based anticancer interventions.

CSCs contribute critically to tumor progression, metastasis, drug resistance, and relapse because of their unique self‐renewal and multi‐lineage differentiation capacities [23, 24, 25]. SOX2, a core TF maintaining stem‐cell pluripotency, sustains tumor stemness and assumes an instrumental role in chemoresistance [26, 27]. Lu et al. [28] reported that the long non‐coding RNA TDRG1 aggravates LC stemness in a SOX2‐dependent manner, whereas SOX2 knockdown reverses CDDP resistance in NSCLC via the APE1 pathway [12]. We observed that CDDP‐resistant NSCLC cells exhibit elevated SOX2 expression and suppressed cuproptosis. Cuproptosis induction is proven to enhance CDDP sensitivity in cervical cancer [29] and SOX2 is a cuproptosis‐related gene in glioma [30]. We thus hypothesized that SOX2‐mediated CDDP resistance might involve cuproptosis. Indeed, as evidenced by experiments, SOX2 silencing markedly inhibited proliferation and induced apoptosis, effects that were effectively reversed by the cuproptosis inhibitor TTM. This finding extends previous knowledge. In CDDP resistance of NSCLC, SOX2 functions primarily through cuproptosis modulation rather than through canonical stemness maintenance. The investigation offers a new perspective on tumor resistance mechanisms.

Mechanistically, SOX2 was significantly positively linked with Wnt/β‐catenin downstream targets. SOX2 overexpression significantly activated Wnt/β‐catenin signaling in CDDP‐resistant cells. This finding is of substantial biological importance on the basis of an established study that shows aberrant Wnt/β‐catenin activation promoting CSC renewal, proliferation, and differentiation and fostering tumorigenesis and therapeutic resistance [31]. As a multifunctional TF, SOX2 has been verified to activate the Wnt/β‐catenin signaling pathway through multiple mechanisms, including directly transactivating Wnt ligands [32] and boosting the nuclear accumulation and transcriptional activity of β‐catenin [33]. In addition, SOX2 facilitates the development of cisplatin resistance via the Wnt pathway [34]. The critical advance of our study is the delineation of the SOX2–Wnt/β‐catenin–ATP7B regulatory axis. Building on previous reports that the β‐catenin/TCF4 complex directly binds the ATP7B promoter to induce its expression [10, 35]. This study found that SOX2 overexpression activates the Wnt/β‐catenin signaling pathway. Moreover, LF3, an inhibitor of the Wnt pathway, significantly reversed the oe‐SOX2‐mediated upregulation of ATP7B. These findings demonstrate that SOX2 regulates ATP7B in a Wnt/β‐catenin activation‐dependent manner, rather than through direct transcriptional regulation of ATP7B by SOX2. Importantly, we newly link this axis to cuproptosis sensitivity: activation of SOX2–Wnt/β‐catenin–ATP7B increases cellular Cu^2+^ efflux, suppresses cuproptosis, and enhances CDDP resistance.

The present findings are confined to A549 cells; future work will establish additional CDDP‐resistant NSCLC models to examine the generality of the SOX2/Wnt/ATP7B axis across NSCLC subtypes. Correlations between this axis and clinical CDDP resistance in human lung specimens will also be investigated. Despite these remaining questions, our work is the first to reveal that SOX2 confers CDDP resistance through the Wnt/ATP7B‐mediated suppression of cuproptosis. This discovery provides new theoretical insight into NSCLC chemoresistance. Moreover, it suggests that specific inhibitors targeting key components of this axis may offer a promising strategy to overcome LC chemotherapy resistance.

## Funding

This work was supported by Natural Science Fund Project of Fujian Province (Grant number: 2024J011610).

## Conflicts of Interest

The authors declare no conflicts of interest.

## Data Availability

The data and materials in the current study are available from the corresponding author on request.
